# Reactivation of latent *Histoplasma* and disseminated cytomegalovirus in a returning traveller with ulcerative colitis

**DOI:** 10.1099/jmmcr.0.005170

**Published:** 2018-11-23

**Authors:** Olivia Lucey, Iain Carroll, Thomas Bjorn, Michael Millar

**Affiliations:** The Royal London Hospital, Whitechapel Road, Whitechapel, London E1 1BB, UK

**Keywords:** progressive disseminated histoplasmosis, cytomegalovirus, lymphohistiocytosis, haemophagocytic, reactivation, immunosuppression, liposomal amphotericin B

## Abstract

**Introduction:**

We describe a case of progressive disseminated histoplasmosis (PDH) and disseminated cytomegalovirus (CMV) with development of haemophagocytic lymphohistiocytosis in a 62-year-old man of Bangladeshi origin living in the UK.

**Case presentation:**

The patient had a background of ulcerative colitis for which he took prednisolone and azathioprine. He presented with fever, lethargy, cough, weight loss and skin redness, and was initially treated for bacterial cellulitis and investigated for underlying malignancy. He developed multiple progressive erythematous skin lesions, sepsis and colitis requiring management on intensive care. A skin biopsy showed yeasts in the dermis and sub-cutaneous fat, which were confirmed as *Histoplasma capsulatum* by PCR. Disseminated CMV with evidence of end organ gastrointestinal disease was also diagnosed. Despite anti-viral and anti-fungal treatment, the patient deteriorated with evidence of bone marrow suppression and a diagnosis of haemophagocytic lymphohistiocytosis was made.

**Conclusion:**

PDH is classically seen in patients with significant immunosuppression, e.g. those with human immunodeficiency virus (HIV) or on anti-TNF therapy; however, we present a case of reactivation of *Histoplasma* in a non-HIV patient. We consider the importance of contemplating reactivation of endemic mycoses and CMV in critically unwell and deteriorating patients.

## Introduction

This case highlights the importance of carefully considering infectious differentials in a rapidly deteriorating patient with sepsis; with specific focus on reactivation of latent infections acquired in endemic regions, either from recent travel or prior exposure at any time. Progressive disseminated histoplasmosis (PDH) is a rare disease that is usually associated with cellular immune dysfunction, particularly in hosts with human immunodeficiency virus (HIV) or on anti-TNF therapy, but should also be considered in patients on ‘lighter’ immunosuppression, such as low-dose prednisolone with a compatible clinical syndrome [1]. The case also demonstrates the usefulness of tissue biopsies in atypical skin lesions in unwell patients with emphasis on rapid diagnostics, e.g. PCR in diagnosing *Histoplasma capsulatum*. Once the diagnosis of haemophagocytic lymphohistiocytosis (HLH) is established, early identification of its driver(s) is crucial. This patient had both evidence of disseminated cytomegalovirus (CMV) infection and histoplasmosis; highlighting the need for clinicians to consider multiple infectious aetiologies in critically unwell patients.

## Case report

A 62-year-old man of Bangladeshi origin was brought to the emergency department with lethargy and fever. Over the previous 2 weeks, he had developed a productive cough and weight loss. His past medical history included ulcerative colitis (diagnosed in 1999), which was quiescent on surveillance colonoscopy 2 days prior to admission. A suspicious rectal lesion was, however, biopsied. He had coronary artery bypass grafting in 1999, type 2 diabetes, hypertension, hyperlipidaemia and chronic hepatitis B. His medication included prednisolone, 10 mg daily (which he had been taking for 15 years), azathioprine 150 mg daily, Asacol (mesalazine) 2 mg daily, tenofovir and allopurinol 100 mg daily. His family acknowledged he had been taking prednisolone at greater than the prescribed dose for approximately 2 months prior to admission.

The patient was born in Sylhet, Bangladesh, and had moved to the UK aged 14. 6 months previous to the admission, he had travelled back to Sylhet for a 1 month family visit. He had not been unwell during the trip or until presentation. There was no unusual exposure history. There was no other significant or relevant travel history during his lifetime.

On examination he had a temperature of 34.8 °C, a non-tender erythematous left calf overlying a saphenous vein harvest site. His haemoglobin was 116 g l^−1^, white cell count 6.2x10^9^ per litre and C-reactive protein 138 mg l^−1^. Initial management included empirical intravenous flucloxacillin 1 g four times daily for presumed cellulitis. A lower limb ultrasound excluded deep vein thromboses, and a computed tomography scan of chest, abdomen and pelvis demonstrated a rectal mass and a right basal pneumonia. Antibiotics were changed to intravenous benzylpenicillin 1.2 g four times daily and oral clarithromycin 500 mg twice daily.

**Fig. 1.**
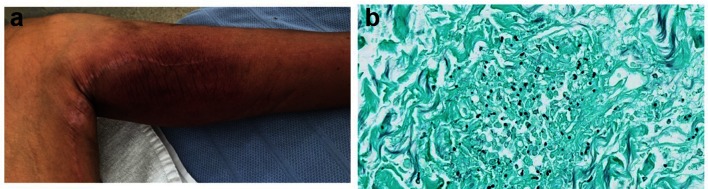
(a) Medial aspect of the left leg. (b) Grocott–Gomori stain of a skin biopsy.

Over the initial week, his condition deteriorated with fever to 40 °C, rising C-reactive protein and erythema spreading up the left leg to the scrotum, abdomen, left flank and right leg. The skin became tender and warm to touch. Dermatologists reviewed the skin on day 6 (Fig. 1a). A differential diagnosis of multifocal cellulitis, panniculitis or migratory thrombophlebitis, in the context of possible malignancy was made. The patient developed a tender, swollen right testicle. An ultrasound showed epididymo-orchitis. Empirical treatment with oral ciprofloxacin 500 mg twice daily and oral doxycycline 100 mg twice daily was added.

The infectious diseases team suggested changing the benzylpenicillin to intravenous ceftriaxone 2 g daily, and adding oral linezolid 600 mg twice daily and intravenous amikacin 1.2 g once daily. Vasculitic, auto-immune and sexual health (HIV, chlamydia, gonorrhoea) screens and mycoplasma serology were negative. A trans-thoracic echocardiogram showed no signs of endocarditis. Blood cultures were negative.

On day 12, the patient was transferred to critical care for ventilation and noradrenaline (dose titrated to blood pressure within the standard critical care range of 6–13 µg min^−1^) for septic shock, considered secondary to cellulitis. He remained febrile at 40 °C and had developed a coagulopathy and bloody diarrhoea. His antibiotics were changed to meropenem 1 g three times daily and linezolid 600 mg twice daily. Plastic surgeons and urologists did not suspect necrotizing fasciitis.

The erythema spread to his flanks. No papules, nodules or umbilicated lesions were noted. In view of the diagnostic uncertainty, a skin biopsy was performed on day 15. A rectal biopsy showed CMV inclusion bodies and the plasma CMV DNA viral load was 15 304 copies ml^−1^. Intravenous ganciclovir 375 mg twice daily was initiated on day 19 to treat suspected disseminated CMV disease with evidence of colitis, epididymo-orchitis and panniculitis. CMV DNA was detected in skin, urine and throat samples.

Due to worsening leucopenia and thrombocytopenia, the ganciclovir was changed to foscarnet 4.5 g three times daily. Fluconazole 400 mg daily was added empirically on day 27 given the lack of clinical improvement, due to the presence of *Candida albicans* in the urine on day 24. The skin biopsy was reported on day 28 as showing septal/lobar panniculitis. To exclude necrotizing fasciitis, a scrotal incision was performed, which was sterile. Examination of the rectum showed no inflammation.

The fluconazole was switched to micafungin 100 mg daily on day 31, due to the growth of *Candida glabrata* from urine culture on day 30. Following a methenamine silver stain, the histopathologists reported the first skin biopsy as showing small, round yeasts of 3–5 microns within well-circumscribed areas of inflammation in the dermis and subcutaneous fat (Fig. 1b). A skin biopsy was repeated the same day with samples sent for microbiological and fungal culture and molecular testing. On day 34, the pan-fungal PCR confirmed DNA from *H. capsulatum*. The micafungin was changed to AmBisome (amphotericin B) 320 mg daily.

A bone marrow biopsy was performed on day 33, which showed pancytopenia with increased macrophage activity, without evidence of malignancy, acid-fast bacilli or fungi. Ferritin and triglyceride levels were raised (47 798 µg l^−1^ and 2.45 mmol l^−1^, respectively) supporting a diagnosis of HLH. Immunosuppression was considered, but due to the patient's extremely poor clinical condition was not given. The bone marrow culture subsequently grew *H. capsulatum*. The patient continued to deteriorate and died on day 36 of admission.

## Discussion

We have described a case of PDH in a 62-year-old man living in a non-endemic area for histoplasmosis. This case demonstrates reactivation of latent histoplasmosis, presumably primarily acquired in Bangladesh. It is postulated that viable organisms persist in the tissues following the primary infection and subsequently reactivate [2]. Patients with cellular immune dysfunction, primary immune deficiency disorders, HIV and those on immunosuppressive therapy, e.g. anti TNF-α, are at particular risk of severe or disseminated forms [1]. In this case, the corticosteroids and azathioprine appear to have been the main risk factor for reactivation; however, it has been shown that although these drugs increase the odds of developing an opportunistic infection in inflammatory bowel disease patients (by a factor of 15), histoplasmosis is a rare infection to occur with these immunosuppressive drugs [3]. The fact that the patient had administered higher than prescribed doses of prednisolone prior to admission may have accelerated and contributed to the reactivation process. Culture of *Histoplasma* from extra-pulmonary sites, e.g. skin, bone marrow, is the gold standard for diagnosing PDH [4]. Previous descriptions of skin involvement have included nodules, abscesses, fistulae and cellulitis with plaques rather than a panniculitis [2]. Suspecting a fungal aetiology of atypical skin lesions and early skin biopsy in cases where there is progression, clinical deterioration or diagnostic difficulty is paramount. The in-house pan-fungal rRNA-PCR allowed a diagnosis to be made within 3 days of biopsy, highlighting the important role of PCR in achieving a timely diagnosis as the bone marrow culture was not available until 29 days after the patient's death. Histopathological examination of tissue (in this case, skin) can reveal the typical 2–4 micron yeast structures of *Histoplasma*; however, this lacks specificity, as these structures may be confused with other fungi [4]. Knowledge of the geographical distribution of *H. capsulatum* is incomplete [5]. Disseminated histoplasmosis has been described in Bangladesh [6]. It is plausible that endemic areas for histoplasmosis are more widespread than commonly documented.

The consequences of CMV reactivation in unwell patients are not well understood, and the subject of debate and research. In immunocompromised critically ill hosts, CMV has an immunomodulatory effect with mortality from secondary infections, malignancy and cardiovascular disease, and the evidence supports prophylaxis and treatment in these settings [7]. In immunocompetent patients, the evidence is less clear; whether CMV acts as a pathogen or a marker of disease severity remains uncertain. Some studies categorize recent corticosteroid use as significant immunosuppression; however, others do not [8]. The most frequent precipitant for reactivation is sepsis, with corticosteroids also recognized [9].

The clinical impact of the disseminated CMV infection in our case is unclear, but is likely to have contributed to the patient’s deterioration. Our patient received 17 days of anti-virals without improvement. It is important to consider the adverse effects of anti-virals; perhaps the bone marrow suppression was potentiated by the ganciclovir. The *Histoplasma* infection is likely to have been the more significant pathogen and precipitating factor for HLH.

We had not considered a fungal aetiology until late in the disease course. Initially a bacterial cellulitis was considered the main differential, despite an atypical distribution of erythema, poor response to empirical antibiotics and negative blood cultures. Subsequently, the disease was attributed to disseminated CMV, prior to the diagnosis of histoplasmosis. We discussed our propensity to biases as clinicians. Anchoring bias occurs when clinicians focus on one diagnosis despite evidence to the contrary, whilst confirmation bias acknowledges only information that confirms or refutes the suspected diagnosis [10]. On reflection, alternative diagnoses should have been considered earlier. The immunosuppressant properties of steroids are perhaps under-estimated in the presence of other factors such as age, co-morbidities and sepsis. This case highlights the potential for reactivation of latent infections in patients with a history of corticosteroid use, relevant travel or in those who are deteriorating or critically unwell.
